# Special Issue: Response of Microbial Communities to Environmental Changes

**DOI:** 10.3390/microorganisms6020029

**Published:** 2018-03-30

**Authors:** Ulrich Stingl

**Affiliations:** UF/IFAS Fort Lauderdale Research & Education Center, Department of Microbiology & Cell Science, University of Florida, Davie, FL 33314, USA; ustingl@ufl.edu; Tex.: +1-954-577-6326

Environmental issues such as eutrophication, ocean acidification, sea level rise, saltwater intrusion, increase in carbon dioxide levels, or rise of average global temperatures, among many others, are impacting and changing whole ecosystems. While changes in plant and animal communities are, in most cases, relatively slow and obvious, alterations in structure and function of microbial communities happen much faster, but mostly stay unnoticed. Microbial communities are clearly the first responders to any environmental change, but details on what exactly happens to community composition or functionality as a response to variations in environmental parameters is in most cases still quite unclear. This lack in knowledge can partly be explained by the fact that microbial communities are extremely complex with thousands of “species”, including many with mostly unknown functions. The responses of microbes to environmental conditions can be measured in situ in whole natural complex communities, in laboratory enrichments with reduced complexity, or in pure cultures or even single cells.

This Special Issue contains seven articles that study the impact of environmental parameters on microbes in three different levels and systems: host–microbe interactions, natural communities, and pure cultures.

Most higher organisms strongly depend on their microbiome to supply them with essential nutrients, protect them from diseases, or to provide them with metabolic capacities that they do not have. Davis et al. [1] report, based on a four-year-long study, that the nitrogen-fixing communities associated with salt marsh plants are quite responsive to drought, but that there are groups of persistent diazotrophs that seem to tolerate and survive these harsh conditions.

Ugarelli et al. [2] report in a review article that there is a lack of knowledge on seagrass-associated microorganisms. Based on the available data, it seems that different parts of seagrasses are colonized by different microorganisms, which most likely reflects different functionality of these communities. One of the main messages is that the sulfur-cycle seems to be very important for seagrasses, as increases in pore-water sulfide concentrations are responsible for many of the recent die-off events. Sulfur-oxidizing bacteria that can convert sulfide to non-toxic sulfate have been reported from the seagrass rhizosphere in several studies.

Nalepa [3] presents a very interesting perspective on the direct impact of host hormones on members of its microbiome. Flagellates are essential for lignocellulose digestion in lower termites and closely related wood-feeding cockroaches. However, while the flagellates in cockroaches are retained during the host’s molting cycle, they die prior to the termites’ ecdysis and have to be re-inoculated in the larvae. By re-analyzing the ground-breaking work by Cleveland from more than 50 years ago, Nalepa provides two alternate hypotheses: either the flagellate symbionts are victims of the change in the host’s social status, or the flagellates have become incorporated into the life cycle of the eusocial termite colony.

Flooding with seawater changes the physico-chemical conditions of coastal areas and thus their microbial communities. Sjøgaard et al. [4] show clearly in six-month-long mesocosm experiments that heterotrophic metabolism and activities in the nitrogen cycle respond the most strongly to this environmental change. Interestingly, the availability of labile organic matter decreased over the duration of the experiment, which was also reflected in changes in the microbial communities.

Wemheuer et al. [5] investigate microbial communities in a transect ranging from the German Bight to the northern North Sea. They show that variations in community composition and function could be explained by the measured environmental properties in the different sampling sites. Commendably, the authors go a significant step further and analyze members of the community that were responsible for the degradation of dissolved free amino acids, adding important missing information on functionality of distinct members of complex marine microbial communities.

Complex communities are, well, complex. Studying single populations in these communities is challenging because of their many known and unknown interactions within the community. If the organism is readily culturable, studies on pure cultures can eliminate this issue and provide a means to test functionality of certain species. Two papers in this issue analyze the impact of environmental parameters on the metabolism of pure cultures.

Zhang et al. [6] show that growth rates of *Carnobacterium maltaromaticum* in vacuum-packaged beef strongly depend on the temperature, atmosphere, and pH as well as on concentrations of lactic acid and glucose. This study has implications for the meat industry as *Carnobacterium maltaromaticum* is non-pathogenic to humans but inhibits growth of other food-spoiling bacteria.

Wasai et al. [7] report that, for carbon-starved cells of *Rhodopseudomonas palustris*, ATP-dependent rearrangement of cells induces increased salt tolerance. As periods of starvation are very common in the marine environment, this study analyzing the impact of the environmental parameters during starvation on the salt tolerance of the cells is an important contribution to the field.
